# Assessing the effectiveness of dialkylcarbamoylchloride (DACC)-coated post-operative dressings versus standard care in the prevention of surgical site infection in clean or clean-contaminated, vascular surgery (the DRESSINg trial): study protocol for a pilot feasibility randomised controlled trial

**DOI:** 10.1186/s40814-019-0400-2

**Published:** 2019-01-18

**Authors:** Joshua P. Totty, Amy E. Harwood, Paris L. Cai, Louise H. Hitchman, George E. Smith, Ian C. Chetter

**Affiliations:** Academic Vascular Surgical Unit, Hull Royal Infirmary, Anlaby Road, Hull, HU3 2JZ UK

**Keywords:** Infection, Surgery, Surgical wound, Dialkylcarbamoylchloride, DACC, Prevention

## Abstract

**Background:**

Surgical site infection in vascular surgery has a reported incidence of up to 19%. A novel method of reducing this rate of infection is dressings coated with dialkylcarbamoylchloride (DACC), a hydrophobic wound contact layer that binds bacteria and removes them from the wound bed. Early research has suggested that DACC-coated wound dressings are effective in reducing surgical site infection when applied to wounds healing by primary intention post-operatively, therefore this trial aims to assess the feasibility of producing high-quality evidence assessing this theory.

**Methods:**

Patients undergoing clean or clean-contaminated vascular surgery will be randomised to have their surgical wounds dressed with a DACC-coated dressing or a non-coated occlusive absorbent post-operative dressing. All other aspects of their peri-operative care will be standardised or carried out in line with hospital policy. Wound assessments will be carried out between day 5–7, day 30 (± 3 days) and 6 months post-operatively (± 7 days) by a blinded assessor using the ASEPSIS scoring tool. Quality of life data using EQ-5D and SF-36, resource use and mortality data will also be collected. This feasibility trial will dictate the conduct of a full-scale trial through the collection of data on recruitment and retention rates, and fitness-for-purpose of the follow-up arrangements.

**Discussion:**

Surgical site infections are now the second most common hospital acquired infections with a significant cost implication. The aim of the DRESSINg trial is to investigate the effectiveness of a novel preventative measure at reducing wound infections post-surgery and will provide robust evidence to support or deny its use.

**Trial registration:**

Clinicaltrials.gov identifier: NCT02992951, Registered 12/12/16. REC Reference: 16/LO/2135.

## Introduction

The Centres for Disease Control and Prevention (CDC) defines a surgical site infection (SSI) as an infection within 30 days of an operation or up to 90 days if an implant is left in place and the infection is related to an operative procedure [1]. SSI’s occur in at least 5% [2] of patients and have a significant impact on patient morbidity, mortality and have subsequent time and cost implications [3].

Microorganisms causing SSI’s may be either endogenous (present on the patient) or exogenous (from the environment). The most common bacteria causing a superficial wound infection are those which are present on the skin of the patient. Deeper wound infections may be due to contamination of the wound by bacteria encountered during a procedure. Despite the reported incidence of SSI’s for clean surgery of 2.1% [4], the incidence in practice varies significantly. Open varicose vein surgery has an incidence of SSI’s reported in the literature which varies from 1.5 to 16% [5, 6] whilst figures from the surgical site infection surveillance demonstrated a high rate of SSI’s in patients undergoing lower limb amputation (13.1%) [7]. Conversely, SSI rates following carotid endarterectomy are reported as low as 0.2% [8, 9].

The Sorbact® range of dressings contains dialkylcarbamoylchloride (DACC), found in a spider’s web, which mediates the irreversible binding of bacteria that exhibit a high cell surface hydrophobicity (CSH). Large numbers of adherent or ‘trapped’ bacteria can then be removed from the wound at each dressing change. They are removed without disrupting the bacterial cell wall, thereby avoiding the resultant increase in inflammation observed with traditional antibiotics or antiseptics [10]. Numerous bacteria and fungi that exhibit CSH have been shown to attach to the DACC-coated material, including *Staphylococcus aureus*, *Pseudomonas aeruginosa*, *Enterococcus faecalis*, *Candida albicans* and the dermatophyte *Trichophyton rubrum* [11, 12].

DACC-coated dressings are available on the open market in both the UK via the National Health Service (NHS) supply chain, and the USA [13].

The current available evidence, although limited, favours the use of DACC-coated dressings in reducing SSI [14]. A recently published randomised controlled trial and preceding pilot study concluded that in a cohort of women undergoing caesarean section in a single centre, DACC-coated dressings proved to be clinically and cost-effective in reducing the rates of SSI [15, 16], indicating that further high-quality studies are needed in this promising area to assess outcomes in a more diverse patient population.

## Methods/design

### Study objectives

The aim of this pilot randomised trial is to evaluate the feasibility of conducting a fully powered randomised controlled trial to investigate the effectiveness of DACC-coated post-operative dressings in the reduction of surgical site infection in vascular surgical patients, using a fraction (one-fifth) of the patients required for a fully powered RCT.

### Study design and setting

This is a pilot feasibility randomised single-centre clinical trial in the setting of a University Teaching Hospital, based in the UK, offering tertiary vascular surgery services. The protocol has been prepared in line with the Standard Protocol Items: Recommendations for Interventional Trials (SPIRIT) guidelines [17].

### Inclusion criteria

The inclusion criteria are:

1. Over 18 years of age
2. Undergoing clean or clean-contaminated vascular surgery (as opposed to contaminated or dirty surgery, in which there is infection already present).
3. Able to understand (i.e. have sufficient language skills) and complete the patient information sheet, consent forms, and questionnaires.
4. Able and willing to give informed consent to participate in the trial (patients may be capacitous but unwilling to give informed consent; they would not meet the inclusion criteria).

### Exclusion criteria

The exclusion criteria are:

1. Patients actively taking antibiotics for other conditions up to the day of surgery (not including surgical prophylaxis or antibiotic use related to the index procedure)
2. Patients undergoing carotid endarterectomy (as these patients have an infection rate significantly lower than rates seen in other types of vascular surgeries [8, 9])
3. Allergies to any component of either the DACC-coated dressing or the control dressing
4. Inability to give informed consent due to incapacity (as defined by the MCA 2005 [18])
5. Aged under 18 years at the time of recruitment
6. Use of investigational drug/device therapy within preceding 4 weeks that may interfere with this study.

### DACC-coated post-operative dressings and post-operative procedures

Patients will receive standardised care pre-operatively. Hair removal (clipping) and anaesthesia will be conducted according to local hospital policy; in our centre, hair is clipped, not shaven, immediately prior to the procedure in the operating theatre. Skin preparation will be standardised to Povidone-iodine in aqueous solution, unless there is a documented patient allergy. Prophylactic antibiotics will be given as per local guidelines and recorded in the patient notes. Patients will be randomised on the day of their surgical procedure to receive post-operative dressing with a DACC-coated dressing (Leukomed® Sorbact®, BSN Medical, Hull, UK), or a non-coated occlusive absorbent control post-operative dressing (standard practice) (OpSite® Post-Op, Smith & Nephew, Hull, UK).

All dressings will be replaced on day 2 post-procedure, and again at the time of first wound review, which takes place between post-operative days 5–7. Interim dressing changes will be permitted where there is a clinical indication such as soiling or damage to the dressing. On discharge from hospital, patients will be provided with further dressings of the same variety to ensure like-for-like dressing changes up to the point of wound healing, as well as instructions to community teams that the only wound dressings to be used are the ones provided for trial purposes, in order to maintain treatment adherence.

### Outcomes

Outcomes will be divided into two sections: *feasibility outcomes* and *clinical outcomes*.

#### Feasibility outcomes

- The measured effect size of the trial intervention, in order to contribute to the power calculation and design of a full scale RCT
- Eligibility rates and reasons for non-eligibility
- Participant recruitment rates and reasons for non-recruitment
- Follow-up and study retention rates and reasons for drop-out/non-attendance
- Fitness for purpose and acceptability of follow-up arrangements
- The suitability of the trial intervention in different wound types/areas
- The suitability of the inclusion/exclusion criteria
- The suitability of outcome assessment measure(s)
- Fitness for purpose and acceptability of data collection methods, including the use of smartphone or online ‘apps.’
- Rates of participant withdrawal from the trial; participant response rates to questionnaires; likely rates of missing study data.

#### Clinical outcomes—primary outcome

The primary outcome will be the incidence of SSI—measured by total ASEPSIS score ≥ 21 [19]—within 30 days of surgery, or as defined by the CDC definition of SSI [1, 20].

#### Secondary outcomes

Secondary outcomes will be:

- The incidence of SSI—total ASEPSIS score ≥ 21 or SSI as defined by CDC definitions—at 90 days for implant patients only (as per the CDC definition for SSI in implant-involving surgery)
- Satisfactory healing—total ASEPSIS score ≤ 10—at 30 days post-surgery for non-implant surgery and implant patients
- Satisfactory healing—total ASEPSIS score ≤ 10—at 90 days for implant patients only
- Quality of life: using EurolQol 5 Domains (EQ-5D-3 L) and Short Form 36 (SF-36) v2 questionnaires at 5–7 days, 30 ± 3 days, 3 months ± 7 days, and 6 months ± 7 days.
- Time to return to normal activity/work (measured in working days between the day of surgery and the day the patient returned to work).
- Resource use—primary care visits, requirement for antibiotics, readmission, re-intervention within 30 days and 6 months.
- 30-day mortality.

### The ASEPSIS scale

The ASEPSIS scale is a standardised tool used to determine the presence of SSI (with a score ≥ 21) or impaired wound healing (score > 10 but < 21) [19]. The ASEPSIS tool has been reported to be repeatable and related to outcome [21, 22]. A score of < 10 is indicative of satisfactory wound healing.

### Quality of life assessments

The EQ-5D questionnaire is a widely recognised and validated generic measure of health-related quality of life. This questionnaire has been assessed for acceptability and validity in a number of patient groups [23, 24]. The SF-36 has been well-validated in a variety of UK populations [25, 26]. It measures eight domains which can be used to calculate summary physical and mental component scores.

### Trial timescales

It is anticipated that the recruitment and treatment phase will be completed within 18 months. The primary endpoint is measured after 30 days, with all patients followed up for 6 months after entry into the trial.

### Sample size calculation

The incidence of SSI’s following clean and clean contaminated, non-implant surgery varies widely between the procedures to be included in this study. Whilst the overall infection rate is quoted as being 2.1–3.3%, this is predominantly based on the findings of Cruse and Foord [27] following their long-term surveillance. This has been criticised as it predominantly relies on questionnaire follow-up, over the phone at day 28 post-surgery. The reality in practice is that the infections rates are much higher and are more frequently diagnosed when examined by a blinded observer [28].

The power calculation is based on the primary endpoint having a 90% power and 5% significance. A non-randomised prospective trial comparing Leukomed® Sorbact® with standard dressings in non-implant, clean or clean-contaminated vascular surgery patients, carried out in the same centre demonstrated an overall reduction in SSI from 19 to 10% [29]. To demonstrate the same reduction in SSI, a sample size of 320 per group, or a total of 712 patients, allowing for a 10% loss to follow-up, is required. Study retention rates will be examined at the first interim analysis, and the power calculation amended accordingly. With such a large sample size, there is a high potential for larger loss to follow-up rates than initially expected. Therefore, this pilot study will aim to recruit one-fifth (144) of the total patients needed. The results of this pilot trial, along with other published data on DACC in the reduction of SSI, will be combined to produce a final power calculation for a full-scale RCT.

### Recruitment

Patients that are referred for a clean or clean-contaminated vascular surgical operation will be considered for the study. Patients may be identified at their initial outpatient appointment, at pre-assessment clinics or on their admission to the ward. Patients admitted on a semi-elective or emergency basis will be identified as they are admitted to the ward. Suitable patients will be made aware of the study and provided with the appropriate information, including the patient information sheet. Following this, patients that express an interest will be invited to a screening visit with a study investigator. For inpatients, this visit may take place on the inpatient ward. At this appointment, patients will be assessed against the inclusion and exclusion criteria, and if potential participants meet said criteria, informed consent to participate in the trial will be taken by a study doctor and recorded on the informed consent form and in the patient notes.

To ensure confidentiality and to adhere to data protection guidelines, participants will be given a unique identifying number which does not allow for the identification of study arms or demographic information. The identifiable patient information will not be available to any person(s) outside of the research group. The patient’s general practitioner will also be informed of study enrolment and study details. No changes to concomitant medications will be made, and no restrictions to trial treatments or methods based on concomitant treatment will be made.

### Randomisation

**Fig. 1:**
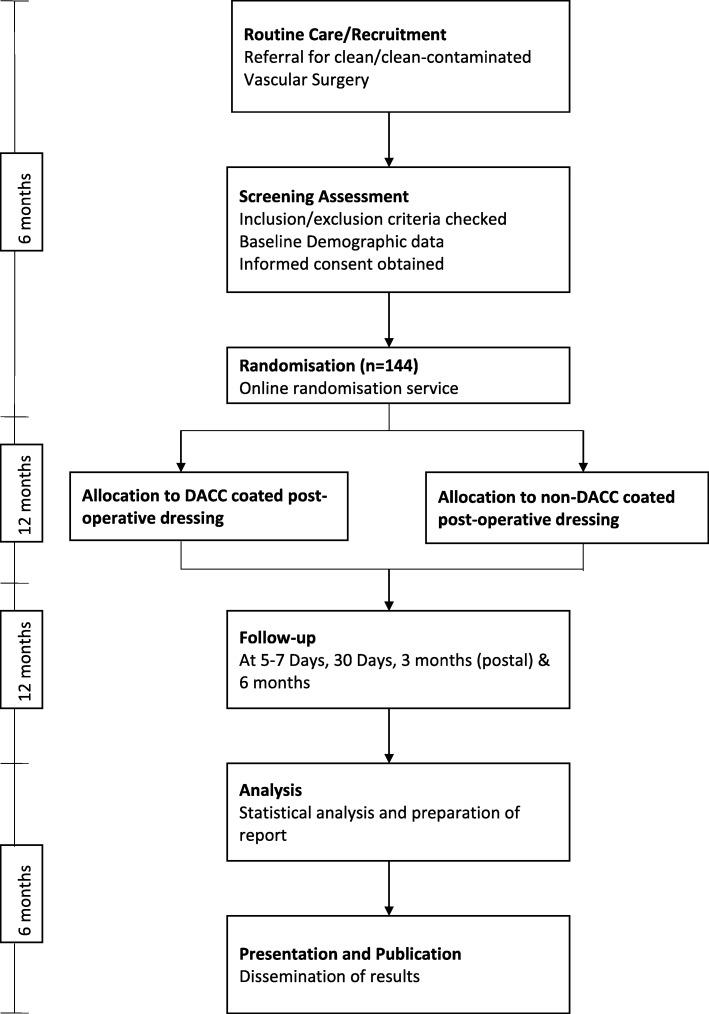
Study flow chart. The target sample size for the pilot phase of this study is 144. Treatment and follow-up will run concurrently, giving a total time of 24 months from study start to completion

Consented patients will be randomised to one of the two parallel testing groups (DACC-coated dressing or standard post-operative dressing) by equal randomisation using the online computerised sealed enveloped method (Sealed Envelope, London, UK) (see Fig. 1).

### Blinding

Due to the nature of the treatment, a double-blind study is not possible (Leukomed® Sorbact® contains a coloured wound contact layer that is not present on standard dressings). To reduce the risk of bias, assessor reported outcomes, namely the ASEPSIS scoring of wounds, will be performed by a blinded assessor who will not have access to the patient notes. Anonymous photos of wounds will be rated by a third blinded assessor to reduce bias and ensure discrepancies in ASEPSIS scores are addressed. Bias in other outcomes will be limited by the use of predetermined objective measurements and bias in quality of life outcomes will be reduced by using patient-reported quality of life outcomes.

### Data collection

All patient data, including patient reported quality of life and clinical outcomes, will be entered into a paper-based case report form (CRF) and anonymised into Microsoft Excel for further analysis and monitored by the research and development department. Only the principal and co-investigators will have access to the full dataset.

### Study visits

Following screening and informed consent, a baseline visit will take place to record demographic data and medical history, prior to randomisation. Study visits will take place between post-op days 5 and 7, at post-op day 30 and at 6 months. Patients will be contacted both by post and telephone when arranging follow-up in order to maximise retention. At the study visits, information such as antibiotic use, length of hospital stay, visits to the general practitioner, dressing changes/number of dressings used and return to work or daily activities will be recorded by a non-blinded study nurse or doctor. Patients will be deemed to have returned to normal activities if they have returned to work (if they are of working age) or if they have returned to their pre-operative activity levels. In particular, patients will be asked if they have returned to driving, exercise or previous levels of social activity.

The same individual will remove the dressing in situ and dispose of it in an opaque bag. A second assessor, blinded to the dressing type, will be invited into the room in order to score the wound on the ASEPSIS scale. This will be recorded in the CRF. With the dressing removed, anonymous photographs will be taken of the wound, to be reviewed by an assessor not involved in trial recruitment or follow-up and used for the standardisation of ASEPSIS scores between participants. With the blinded assessor out of the room, the first study nurse or doctor will replace the dressing. Postal quality of life questionnaires will be sent to participants at 3 months and 6 months. Table 1 outlines the schedule of assessments.

**Table 1:** Schedule of assessments (SPIRIT figure)

Visit	1	2	3	4	5	–	–
	Screening	Baseline assessment	Day 0- day of surgery	Day 5–7 post op	Day 30 post op ± 3 days	3 Months post-op ± 7 days	6 Months post-op ± 7 days
Screening questionnaire	X						
Patient information sheet	X						
Informed consent		X					
Baseline questionnaire		X					
SF-36		X		X	X	X	X
EQ-5D-3 L		X		X	X	X	X
GP letter		X					
RANDOMISATION FORM			X				
HPA surgical wound healing post discharge questionnaire					X		x
Clinical review of wound				X	X		X

*SF36* short form 36 utility, *EQ*-*5D* EuroQoL 5 domain utility index, *HPA* health protection agency

### Trial exit

Patients will exit the trial after completing the 1-year follow-up visit, if they experience an adverse event or reaction deemed to require their withdrawal from the trial or if they choose to withdraw at any point during their inclusion in the trial.

### Statistical analysis

Data will be analysed on an intention to treat basis. The SPSS (IBM Corporation, Armonk, NY, USA) computer package will be used with a two-sided *p* value of < 0.05 taken as the level of significance. For feasibility outcomes, simple categorical data will be presented descriptively using mean (SD), median (IQR) for skewed data or *n* (%) for each group. Recruitment of a large trial within 18 months (based on rates observed in the pilot trial), and retention of 85% or better will be deemed acceptable.

Descriptive statistics (mean (sd) and *n* (%)) will be calculated for demographic and baseline characteristics of the patients. For the clinical outcome of SSI at 30 days, logistic regression analysis will be undertaken with SSI as the dependent variable and randomisation group as the independent variable. The model will be adjusted for confounding variables such as sex, age, BMI, smoking status, diabetes status and surgical site, as well as implant vs non-implant surgery. The regression model performance will be assessed by the Hosmer and Lemeshow test. Logistic regression will also be undertaken for satisfactory healing—total ASEPSIS score ≤ 10. For quality of life (EQ-5D-3 L and SF-36), an intragroup and intergroup analysis will be performed, using Friedman’s two-way analysis of variance (ANOVA) test to assess for intragroup differences, and Mann-Whitney *U* tests to assess for intergroup differences of the SF-36 responses. For the EQ-5D, responses will be dichotomised into ‘no problems’ and ‘problems’, and intragroup analysis conducted using related sample’s Cochrane’s Q test, with Pearson’s χ^2^ tests for intergroup analysis. For time to event data (time to return to work and mortality), Kaplan-Meier and long rank tests will be used to calculate and compare event rates between groups, followed by a cox regression to adjust for confounding variables.

### Monitoring, safety and quality control

The study will be monitored in accordance with the local research and development department’s standard operating procedures to ensure compliance with The International Council for Harmonisation of Technical Requirements for Pharmaceuticals for Human Use (ICH) Good Clinical Practice (GCP) [30] and the Research Governance Framework 2005.

The collection and reporting of data on adverse events and serious adverse events will be in accordance with ICH GCP and the Research Governance Framework 2005. An adverse event (AE) is any untoward medical occurrence in a subject to whom a medicinal product or device has been administered as part of a research study, including occurrences which are not necessarily caused by or related to a medicinal product or device. An adverse reaction (AR) is any untoward and unintended response in a subject to a medicinal product or device. An adverse event becomes serious (SAE) if it results in death, is life-threatening, requires hospitalisation or prolongation of existing hospitalisation, results in persistent or significant disability or incapacity, is a congenital anomaly or birth defect or is otherwise considered medically significant by the investigator. The term ‘life-threatening’ refers to an event in which the patient was at risk of death at the time of the event; it does not refer to an event which hypothetically might have caused death if it were more severe.

Hospitalizations planned prior to enrolment in the trial (elective surgery) or for social reasons should not normally be considered as SAEs unless the hospitalisation has to be prolonged.

The AE reporting period for this trial begins as soon as patients have consented to the trial and ends at the point of discharge from the trial, following a final study visit or telephone consultation at 1 year following surgery. The health status of subjects will be checked at each study visit. The investigator will record all directly observed AEs and all AEs spontaneously reported by the trial subject. A pre-existing condition (i.e. a disorder present at the baseline study visit and noted on the baseline medical history/physical examination form/medical notes) is not to be reported as an AE unless the condition worsens or episodes increase in frequency during the AE-reporting period. All adverse events (serious and non-serious) will be recorded by the investigator in patient’s data collection forms (CRFs) using R&D’s adverse event report form. All adverse events will be recorded by the investigator in patients’ medical records/notes. All AEs will be followed-up by investigators until the event has resolved or a decision has been taken for no further follow-up.

If a clinically significant abnormal laboratory value occurs, this abnormality will be recorded as an adverse event/reaction.

The investigator will report fatal or life-threatening SAEs or serious adverse reactions (SARs) to the research ethics committee (REC) within 7 days and follow-up information within a further 8 days by following the request on the serious event initial and follow-up report forms. The investigator will send all other SAE or SAR reports to the REC within a maximum of 15 days.

Patients have access to information on the complaints procedure for obtaining compensation following harm through non-negligence or negligence as a result of participating in the trial.

Study completion refers to the date of final data collection from the last patient. Paper records from the trial will be stored for 5 years from trial end.

An end of study declaration form will be submitted to the REC and Trust R&D within 90 days from completion of the trial and within 15 days if the trial is discontinued prematurely. A summary of the trial report/publication will be submitted to the REC and Trust R&D within 1 year of the end of trial.

## Discussion

Surgical site infections are now the second most common hospital acquired infections, and cost many thousands of pounds per patient per infection [31]. Any strategy for reducing this burden should be investigated. DACC-coated dressings show no evidence of antimicrobial resistance and have no documented cases of adverse reaction. The aim of the DRESSINg trial is to investigate the effectiveness of this novel preventative measure at reducing wound infections post-surgery.

## Trial status

The DRESSINg trial pilot phase began recruiting in January 2017 and is currently in active recruitment.

## Abbreviations

AE: Adverse event
AR: Adverse reaction
CRF: Case report form
CSH: Cell surface hydrophobicity
DACC: Dialkylcarbamoylchloride
EQ-5D: EuroQol 5 domains
GP: General practitioner
HPA: Health protection agency
HYMS: Hull York Medical School
ICF: Informed consent form
MCA: Mental Capacity Act
R&D: Research and development
REC: Research ethics committee
SAE: Serious adverse event
SAR: Serious adverse reaction
SSI: Surgical site infection
UK: United Kingdom
